# Prenatal Exposure to a Maternal High-Fat Diet Affects Histone Modification of Cardiometabolic Genes in Newborn Rats

**DOI:** 10.3390/nu9040407

**Published:** 2017-04-20

**Authors:** Bijaya Upadhyaya, Tricia Larsen, Shivon Barwari, Eli J. Louwagie, Michelle L. Baack, Moul Dey

**Affiliations:** 1 Department of Health and Nutritional Sciences, Box 2203, South Dakota State University, Brookings, SD 57007, USA; bupadhyaya2@unl.edu (B.U.); shivon.barwari@jacks.sdstate.edu (S.B.); 2 Children’s Health Research Center, Sanford Research, Sioux Falls, SD 57104, USA; Tricia.Larsen@SanfordHealth.org; 3 Sanford School of Medicine-University of South Dakota, Sioux Falls, SD 57105, USA; Eli.Louwagie@sanfordhealth.org; 4 Children’s Health Specialty Clinic, Sanford Children’s Hospital, Sioux Falls, SD 57117, USA

**Keywords:** cardiometabolic disease, chromatin-immunoprecipitation sequencing, developmental programing, histone modifications, maternal high-fat diet

## Abstract

Infants born to women with diabetes or obesity are exposed to excess circulating fuels during fetal heart development and are at higher risk of cardiac diseases. We have previously shown that late-gestation diabetes, especially in conjunction with a maternal high-fat (HF) diet, impairs cardiac functions in rat-offspring. This study investigated changes in genome-wide histone modifications in newborn hearts from rat-pups exposed to maternal diabetes and HF-diet. Chromatin-immunoprecipitation-sequencing revealed a differential peak distribution on gene promoters in exposed pups with respect to acetylation of lysines 9 and 14 and to trimethylation of lysines 4 and 27 in histone H3 (all, false discovery rate, FDR < 0.1). In the HF-diet exposed offspring, 54% of the annotated genes showed the gene-activating mark trimethylated lysine 4. Many of these genes (1) are associated with the “metabolic process” in general and particularly with “positive regulation of cholesterol biosynthesis” (FDR = 0.03); (2) overlap with 455 quantitative trait loci for blood pressure, body weight, serum cholesterol (all, FDR < 0.1); and (3) are linked to cardiac disease susceptibility/progression, based on disease ontology analyses and scientific literature. These results indicate that maternal HF-diet changes the cardiac histone signature in offspring suggesting a fuel-mediated epigenetic reprogramming of cardiac tissue in utero.

## 1. Introduction

Diabetes complicates an estimated 17.8% of all pregnancies in the U.S. [1,2]. Its rising incidence parallels an increasing trend in obesity, as 34.4% of US women of childbearing age are obese [3]. Infants born to women with diabetes or obesity are at higher risk of cardiovascular disease (CVD) at birth and throughout life [4,5,6,7], purportedly through fuel-mediated influences on the developing heart. Both diabetes and hyperlipidemia influence cardiometabolic health in adults, playing a pivotal role in the pathophysiology of CVD over time [8,9,10]. Using a rat model, we have already demonstrated that in utero exposure to diabetes, especially in conjunction with a maternal high-fat (HF) diet, impairs cardiac function through metabolic disturbances in the developing fetal heart [11]. The objective of this study was to determine if exposed offspring from our model have cardiac-specific epigenetic marks that could help explain mechanisms of developmentally-programmed CVD.

The influence of genetic susceptibility and nutritional environment in utero are critical in shaping the metabolic fate of offspring for the rest of their lives [12,13,14]. Mounting evidence demonstrates the importance of transcriptional regulation in normal heart development [15] and metabolic health into adulthood [16]. The heart is the first embryonic organ to function, already beating by three weeks of gestational age [17]. The fully developed heart beats over 100,000 times per day, thus requiring exquisite energy efficiency under a variety of workloads and metabolic circumstances (e.g., aerobic, anaerobic, rest, exercise, or starvation) [18]. For this reason, the heart is an ‘omnivore’, using all types of fuels to make up to 35 kg of adenosine triphosphate (ATP) per day to support normal circulation [19]. Cardiac metabolism changes with development. Given the relatively low levels of oxygen and the readily available fuel supply of the in utero environment, the developing heart favors glycolytic metabolism, in contrast to the adult resting heart, which favors fatty acid oxidation [18,20]. The ability to undergo cardiometabolic switching is essential to long-term heart health. In diabetic and/or obese adults, increased circulating levels of metabolic fuels and impaired insulin sensitivity may lead to changes in cardiac bioenergetics that result in diabetic cardiomyopathy [10]. We have previously shown that a maternal diet that is high in fat, especially in conjunction with maternal diabetes, induces similar lipotoxic effects in the developing offspring’s heart [11,20]. These findings underscore the critical need for research that helps to understand the underlying mechanisms involved in fuel-mediated developmental programming of cardiometabolic disease. 

Over-nutrition during the prenatal period can induce epigenetic changes to alter chromatin structure and influence lasting metabolic health [16]. Specifically, epigenetic processes are a central underlying mechanism in the developmental origin of type 2 diabetes and CVD [21]. Two major types of epigenetic marks, DNA methylation and posttranslational modifications (PTMs) of histone tails, crosstalk with each other and lead to gene expression changes [22]. In particular, environmentally inducible as well as inheritable PTMs may be involved in both gene activation and repression [14] by changing the chromatin state (open or closed), which directly affects the accessibility of promoter regions for the initiation of transcription [23]. The existence of a cardiac-specific, genome-wide histone signature in offspring exposed prenatally to a maternal HF diet, alone or alongside maternal diabetes, has not been established. To investigate this, we used our rat model [11] to delineate cardiac-specific histone modifications associated with genes involved in cardiometabolic functions. Specifically, we investigated the effect of in utero exposure to a maternal HF diet in conjunction with streptozotocin-induced, late-gestation diabetes on four major histone PTMs in neonatal cardiac tissue. We report genome-wide changes in gene-activation and gene-suppressive histone signatures and identify associated genes and quantitative trait loci (QTLs) that are linked to metabolic functions. 

## 2. Materials and Methods 

### 2.1. Animal Study and Dietary Intervention 

**Figure 1:**
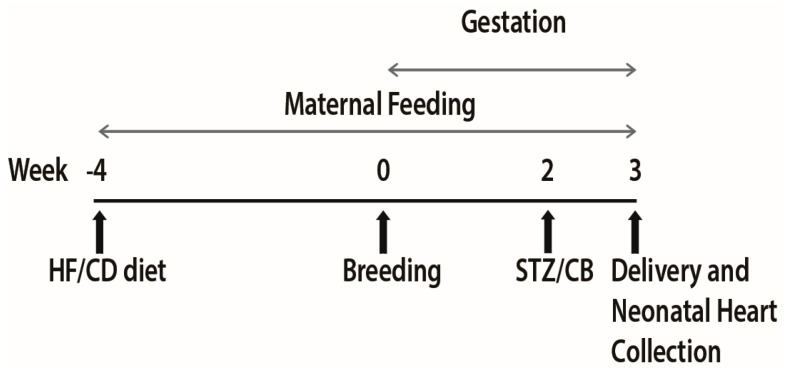
Study timeline. Female rats were randomized into four groups. Two groups were fed high fat diet (HF) and two control diet (CD) for 28 days prior to their breeding and throughout the pregnancy. On gestational day 14, one each of HF and CD group received intraperitoneal injection of Streptozotocin (STZ, 65 mg/kg) and the remaining two received citrate buffer (CB) as a control. Pups were normally delivered on gestational day 22 and sacrificed for heart collection.

Animal work followed the guidelines set forth by the Animal Welfare Act and the National Institutes of Health Guide for the Care and Use of Laboratory Animals and was under approval from the Sanford Research Institutional Animal Care and Use Committee. The protocol titled “Cardiovascular effects of late gestation hyperglycemia and high fat diet in offspring of diabetic mothers: Are lipids the culprit?” was approved on 23 September 2014 (93-08-17B). Methods and model characteristics are reported in detail [11,24]. In short, female Sprague-Dawley rats (Harlan Laboratories, Indianapolis, IN, USA) received a control diet (CD) with 18% of calories as fat (TD2018 Teklad; Harlan Laboratories, Madison, WI, USA) or HF diet with 40% of calories as fat (TD95217 custom diet Teklad; Harlan Laboratories) for at least 28 days prior to breeding. Females were bred with normal, CD-fed males. Gestational day 0 was designated by a positive swab for spermatozoa. On gestational day 14, dams received either 0.09 M citrate buffer (CB) placebo or 65 mg/kg of intraperitoneal streptozotocin (STZ) (Sigma Life Sciences, St. Louis, MO, USA) to induce diabetes (Figure 1). Thereafter, blood glucose levels were kept between 200–400 mg/dL and ketones were kept to a minimum through monitoring and twice daily sliding scale insulin administration. Offspring delivered normally on gestational day 22. Newborn rat hearts were collected from four different groups: controls (control diet and citrate buffer exposed or CC), diabetes-exposed (control diet and STZ exposed or CS), diet-exposed (high fat and citrate buffer exposed or HC), and combination-exposed (high fat and STZ exposed or HS). Hearts were snap frozen and stored at −80 °C until analyses.

### 2.2. Chromatin Immunoprecipitation (ChIP) Assay

Chromatin immunoprecipitation followed by sequencing (ChIP-seq) was performed to generate extensive genome-wide data sets profiling four selected histone modifications across the four exposure groups. Frozen hearts (3–4 from litter mates) were pooled (~250 mg) and homogenized followed by chromatin isolation using P-2001 ChromaFlashTM Chromatin Extraction Kit (Epigentek, Farmingdale, NY, USA). Chromatin was sheared using Episonic2000 Sonication System (Epigentek, Farmingdale, NY, USA) in 300 μL of ChIP buffer, followed by a quality control of sheared chromatin using fluorescence quantification. A minimum of 4 μL of sheared chromatin was purified to obtain DNA, which was eluted with 20 μL of water. DNA fragment (100–300 bp) was quality-checked using a bioanalyzer. For each ChIP reaction, 10 μg of chromatin, 3 μg of histone 3 trimethylated at lysine 4 (H3K4me3) polyclonal antibody or 3 μg of histone 3 trimethylated at lysine 27 (H3K27me3) antibody or 3 μg of histone acetylated at lysine 9 and 14 (H3K9/14ac) polyclonal ChIP-grade antibody (all antibodies were from Epigentek, Farmingdale, NY, catalogue numbers are: A4021, A4039, A4033) and 6 μL of protein A/G beads were used. ChIPed deoxyribonucleic acid (DNA) was eluted in 20 μL of water. Non-ChIPed DNA was used as input controls for the test groups. Since acetylation is a gene-activating mark irrespective of the associated lysine residue and the same antibody targeted both residues, observed acetylation peaks for lysine 9 and 14 are presented together and are referred to as H3Ac, hereafter. Therefore, a total of 12 biological datasets plus four input control datasets were generated. 

### 2.3. ChIP Sequencing

A ChIP-seq library was prepared using the EpiNext ChIP-Seq High Sensitivity Kit with amplification, DNA end polishing and adaptor ligation, following the manufacturer’s instruction. 10 nM of sample libraries were provided for next generation sequencing on a HiSeq 2500 (sequencing was carried out by Epigentek Inc., Farmingdale, NY, USA). The basic analysis of ChIP-Seq was based on the published protocol [25] utilizing Bowtie, version 1.0.0 [26] and MACS, version 2.1.0 [27]. Raw reads were quality checked using FastQC, version v0.10.1 [28] and mapped onto the rat RN5 genome sequence using Bowtie, version 1.0.0 [26]. The option of “-m1” was activated so that only uniquely mapped reads were allowed to map. The mapping results in SAM were converted to BAM and sorted according to coordinates using samtools, version 0.1.19 [29]. Mapping results of each ChIP sample and the input sample were subjected to ChIP enriched peak calling. The option of “-broad” was activated to optimize the calling algorithm for broad binding regions. The called peaks were annotated to the nearest TSS (Transcript Starting Site) using ChIPpeakAnno, version 3.2.0 [30]. ChIP quality control was performed using ChIPQC, version 1.2.2 [31]. Raw nucleotide sequences are available in the National Center for Biotechnology Information (NCBI) gene expression omnibus database under the accession number GSE84831. 

### 2.4. Bioinformatics Analyses

Enriched peaks were called and then annotated to the nearest transcription start site of the genes. Peaks were visualized using Integrative Genomics Viewer [32]. Genomic distribution of peaks were summarized in a heat map using Seqminer version 1.3.3 [33]. First, the genomic reference peak set (TSS.rat.Rnor_5.0.bed) was imported. Then the mapping results (BAM) were loaded; read densities were extracted and clustered (linear Kmeans clustering) using the default parameters. Differential binding analyses was carried out to determine the differential enrichment of cardiac-specific genes due to four different diet-conditions in all regions and also in the promoter region specifically. Downstream validation was carried out for the promoter regions by identifying CpG islands using online database [34] followed by a gene ontology analysis [35] to determine which biological processes were triggered by the four different diet/diabetes conditions using online database GOEAST [36]. The rat genome database [37] was extensively used to annotate the gene, overlapping quantitative trait loci (QTL) and disease ontologies. Briefly, the disease association of each gene in the genome was derived using Disease Ontology and peer-reviewed evidence from GeneRIF [38]. A condensed version of the Disease Ontology, Disease Ontology Lite, was used for the statistical analysis [39].

### 2.5. RNA Isolation and RT-PCR

Rat hearts were homogenized using Precellys 24 lysis and homogenization system (Bertin Technologies, Rockville, MD, USA). RNA was extracted using the RNeasy Fibrous Tissue Mini kit (Qiagen, Germantown, MD, USA) according to protocol. Ribonucleic acid (RNA) integrity was assessed by electropherograms via 2100 BioAnalyzer (Agilent Technologies, Santa Clara, CA, USA) and demonstrated RNA Integrity Numbers (RINs) from 9.7 to 10. RNA concentrations and absorbance ratios were measured utilizing an Epoch spectrophotometer (BioTek, Winooski, VT, USA). Average 260/280 ratios were 2.10 with a range from 2.023 to 2.124. RNA concentration ranged from 645 to 2115 ng/µL. Thereafter, 1 μg of RNA was used to synthesize cDNA using the iScript cDNA synthesis kit (BioRad, Hercules, CA, USA). Quantitative PCR was performed using a standard TaqMan approach performed in ABI7500 qPCR system using the Absolute Blue qPCR Mix (ThermoFisher, Waltham, MA, USA). Beta-2-microglobulin was used as a reference gene for normalizing gene expression levels. Probe/primer sets were obtained from Integrated DNA Technologies (Coralville, IA, USA) and ThermoFisher (Waltham, MA, USA). 

### 2.6. Statistical Analyses 

We compared how histone landscape differs among pups born to a healthy dam with those born to a diabetic or HF-diet exposed pregnancy. We also evaluated a combined effect of GDM and HF exposure in utero. Statistical analyses were performed with Sigma Plot (Systat Software, Inc., San Jose, CA, USA). To compare four exposed-groups, a two-way ANOVA followed by a post-hoc Tukey’s test was performed. When interaction was significant, one-way ANOVA with Tukey’s HSD test was performed. Principal coordinate analysis was used to show the difference among three histone modifications. The genome mapping statistics were extracted using Picard, version 1.90 [40]. Differential binding analyses were carried out using a DiffBind Bioconductor R package [41]. The differential binding comparisons use two matrices at a time where one matrix is fixed and the other one varied between the two groups being compared. For double exposed (HS) pups, we considered diabetes exposed pups (CS) as the comparator to account for the effects of HF-diet in the presence of diabetes. Significance of the association of the mapped histone peaks in gene ontology and disease ontology were determined using Fisher’s exact test. Data were expressed as mean ± SEM, unless otherwise mentioned. A value of *p* < 0.05 or FDR (false discovery rate) <0.1 was considered significant.

## 3. Results

### 3.1. Maternal Late Gestation Diabetes Increases Heart Weight in Offspring

**Figure 2:**
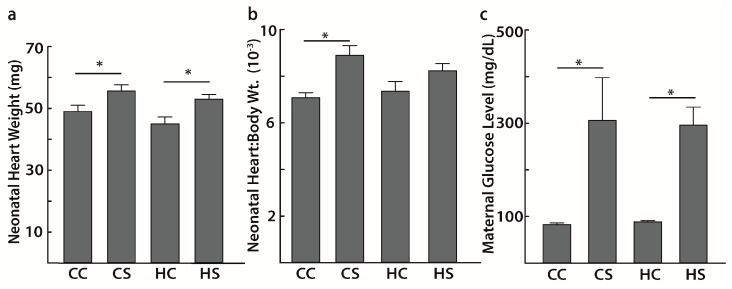
Physiological characteristics of pups and corresponding mothers. Higher neonatal heart weight (**a**) and neonatal heart to body weight ratio (**b**) that correspond to elevated maternal late gestation glucose levels (**c**) in diabetes exposed group; *n* = 10–12 (**a**,**b**), *n* = 2 (**c**). * *p* < 0.05, CC, controls; CS, diabetes exposed; HC, HF-diet exposed; HS, combination exposed.

We compared the average weight of the neonate hearts that were subjected to ChIP-seq experiments. Similar observations were previously reported with echocardiographic data in larger cohorts of rat pups using the same animal model (Figure 1) [11,24]. Although we did not observe any significant interaction effect on neonatal heart weight due to combined diet and diabetes exposure, diabetes-exposed (CS) pups had significantly higher average heart weight compared to controls (CC) (55.64 ± 1.95 mg vs. 48.94 ± 2.03 mg, *p* < 0.05). A similar effect was observed in combination-exposed (HS) pups when compared with the diet-matched controls (HC) (52.92 ± 1.62 mg vs. 44.91 ± 2.30 mg, *p* < 0.05) (Figure 2a). Diabetes-exposed pups also had a significantly higher heart to body weight ratio when compared to controls (0.0089 ± 0.0004 vs. 0.0071 ± 0.0002, *p* < 0.05) (Figure 2b). These data corresponded to elevated maternal late gestation glucose levels induced by STZ when compared to their respective CC and HC diet-matched control groups (305.30 ± 92.01 mg/dL vs. 82.23 ± 3.92 mg/dL and 295.42 ± 39.27 mg/dL vs. 88.30 ± 2.61 mg/dL; both *p* < 0.05) (Figure 2c). We did not observe any significant interaction effect on maternal late gestation glucose levels due to combined exposure. 

### 3.2. In Utero Exposure to Diabetes or HF-Diet Associates with Differential Histone Modifications in Offspring Cardiac Tissue 

**Figure 3:**
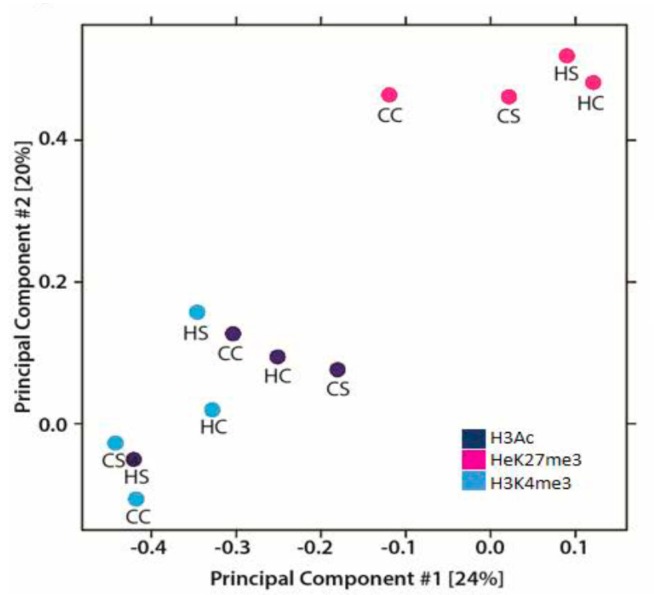
Differential histone modifications. Principal component analyses showing different profiles among three histone modifications (H3Ac, H3K4me3, and H3K27me3) and the four exposure groups. Axes showing % of variations. CC, controls; CS, diabetes exposed; HC, HF-diet exposed; HS, combination exposed.

**Figure 4:**
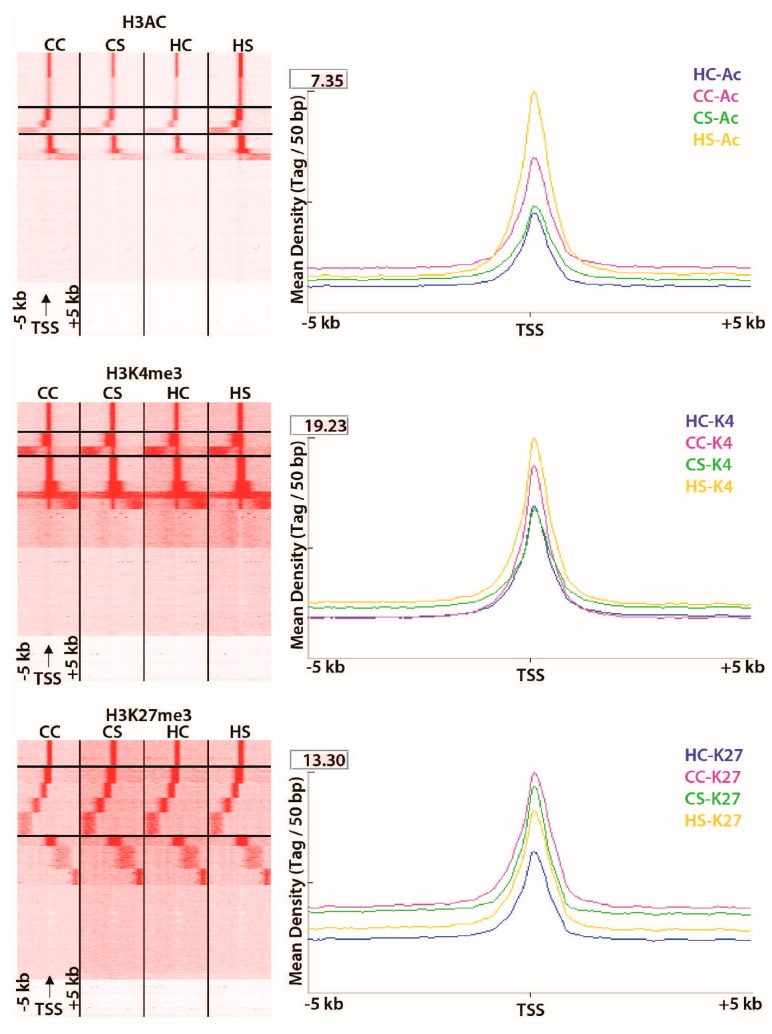
High fat and diabetes-induced genome wide differential histone marks. Heat maps showing read density along with corresponding mean density (tag/50 bp) plots in ±5 kb of any known transcriptional start site for H3Ac, H3K4me3, and H3K27me3 modifications clustered and visualized as four panels in the order of CC (control), CS (diabetes exposed), HC (diet exposed), and HS (combination exposed) from left to right. From the vertical view, clusters with the central density profile are located at the top, followed by clusters with peaks in the −5 kb region (marked within the black lines), those with peaks in the +5 kb region, those with diffuse read density, and those without significant read density.

**Figure A1:**
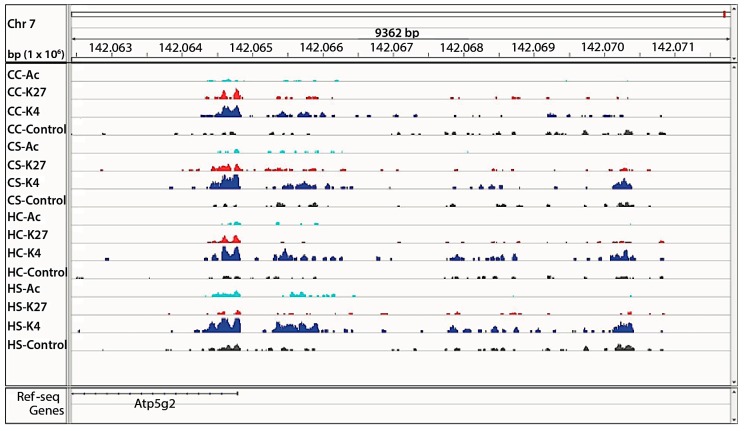
Peaks detection in ChIP sequencing: Representative genomic region of a negative strand (chromosome 7) near the transcriptional start site of Atp5g2 gene, illustrating 5 kb upstream peaks in cyan (acetylation), red (H3K427me3), blue (H3K4me3), and grey (Input control) colors. CC, controls; CS, diabetes exposed; HC, HF-diet exposed; HS, HF-diet and diabetes exposed or combination exposed.

**Figure A2:**
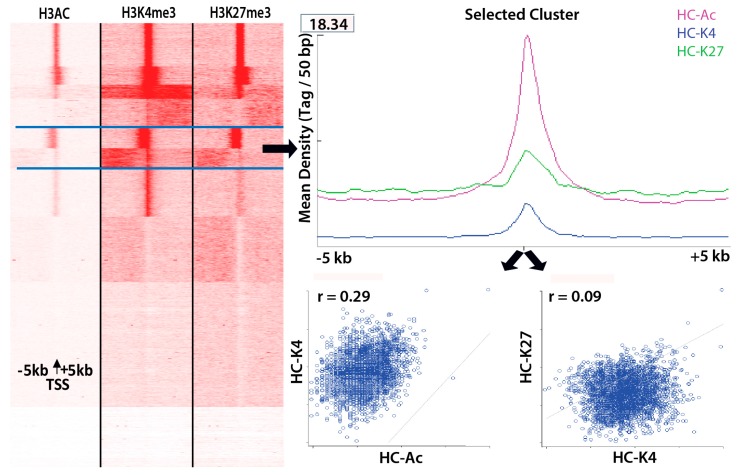
Genome wide differential histone marks in H3Ac, H3K4me3, and H3K27me3 modifications. Read density in ±5 kb of any known transcriptional start site for each marker are clustered and visualized in a heat map that consists of three panels in the order of Ac, K27, and K4 (from left to right). A selected cluster with differential density profile centralized at the transcription start sites are marked under the blue lines, which shows a slight correlation (Pearson coefficient, *r* = 0.29) between K4 and Ac modifications while no correlation (Pearson coefficient, *r* = 0.09) is observed between K4 and K27 modifications due to maternal high fat( HF) diet. HC, HF-diet exposed treatment.

**Figure A3:**
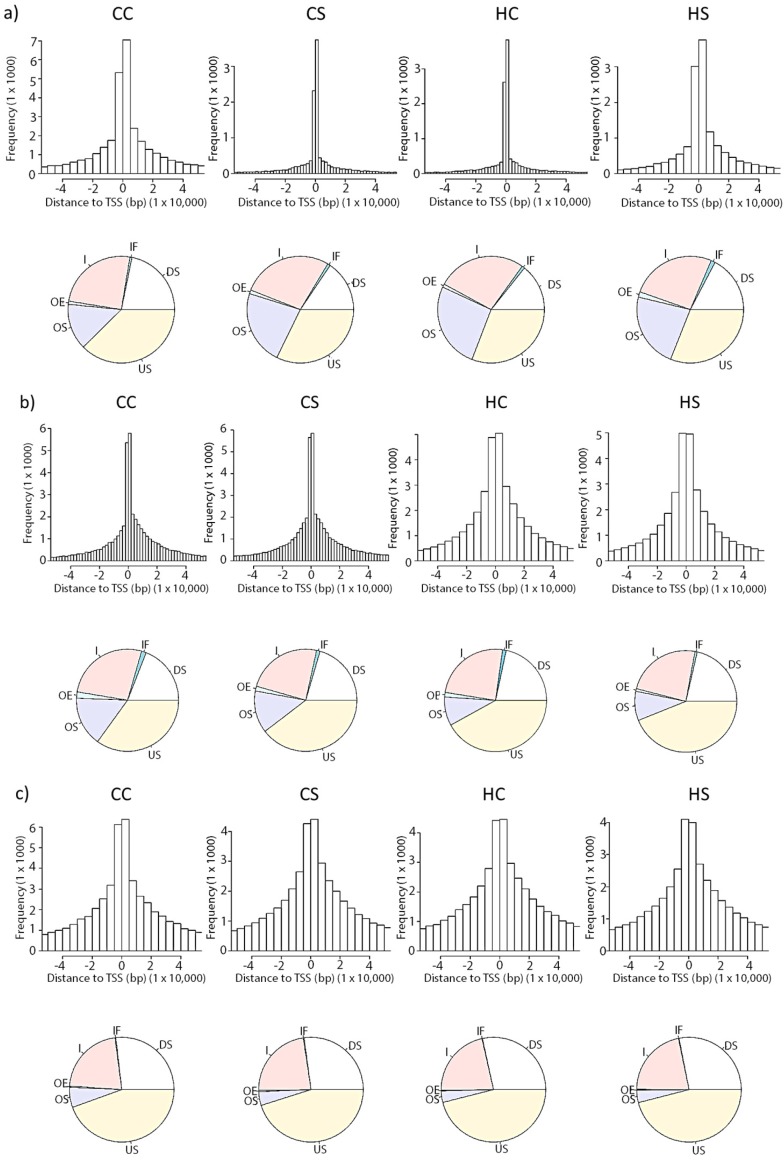
Genic distributions of differential histone marks. (**a**) H3Ac, (**b**) H3K4me3, and (**c**) H3K27me3 in four diet groups around the TSS in four diet groups are plotted. Distances to the nearest TSS for peaks identified and plotted in pie charts. CC, controls; CS, diabetes exposed; HC, HF-diet-exposed; HS, diet and diabetes exposed or combination exposed; TSS: transcription start site; US: upstream; DS: downstream; IF: include feature; I: inside; OE: overlap end; OS: overlap start.

ChIPed DNA sequence revealed differential peak distribution among the diet groups. The highest concentration of enriched peaks (around 40% enrichment compared to input DNA) were overlapping or upstream (−5 kb or gene promoters) of the transcription start site (TSS) for all three modifications in the treatment groups (Figure A1, Figure A2 and Figure A3). About 29, 11, and 36% of approximately 41,000; 73,000; and 54,000 enriched peaks for H3Ac, H3K27me3, and H3K4me3 respectively overlapped to known exonic regions when mapped to the rat genome (data not shown). The genome occupancy of the gene-activating histone marks (H3Ac, H3K4me3) were distinct from the suppressive mark H3K27me3 by 24% and 20% on axes 1 and 2, respectively (Figure 3). The 5 kb upstream regions in the combination-exposed newborn heart genome had relatively higher concentrations of gene-activating marks (H3Ac and H3K4me3) and lower concentration of repressive marks (H3K27me3) as measured in terms of mean peak densities (tag/50 bp) compared to diabetes or diet-exposed hearts as shown by the Seqminer density plots (Figure 4). In the diet-exposed group, the two gene-activating marks, H3Ac and H3K4me3, showed small but higher association between each other (Pearson’s *r* = 0.29) than between the gene-activating and gene suppressive methylation marks (Pearson’s *r* = 0.09) (Figure A2). Together, the effect of the diabetes and HF diet affected the gene-upregulatory histone marks in a way distinct from its influence on the gene-suppressive H3K27me3 mark.

### 3.3. Differential Binding Analyses of Diabetes and Diet Induced Histone Modifications 

**Figure 5:**
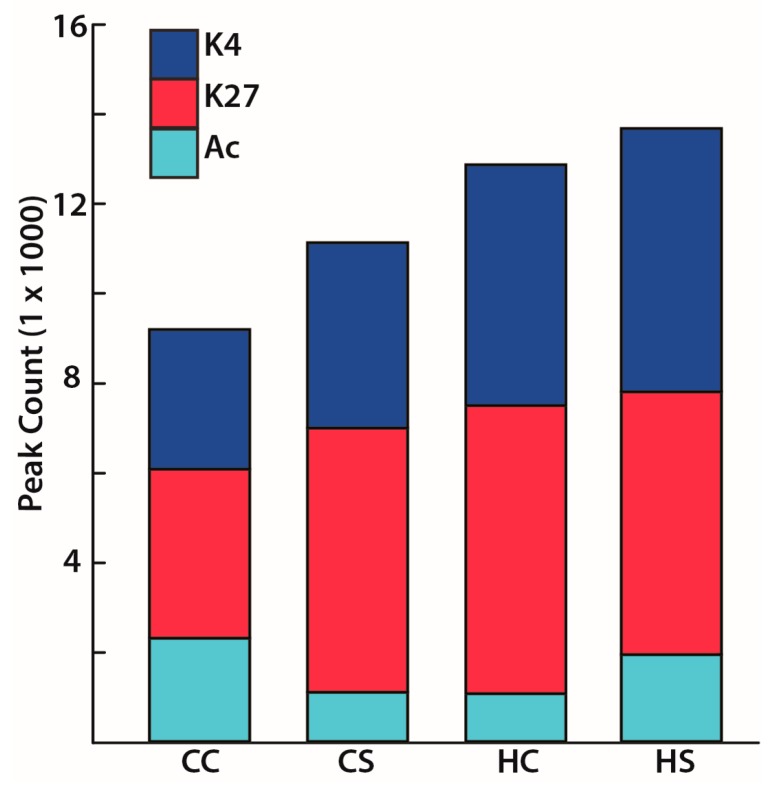
Enriched histone modification peaks overlapping 5 kb upstream region of known transcriptional start site. All H3Ac (Ac), H3K4me3 (K4), and H3K27me3 (K27) enriched peaks detected on the rat genome are shown for each exposure group. CC, controls; CS, diabetes exposed; HC, HF-diet exposed; HS, combination exposed.

Mapping the differential histone modifications to the annotated rat genome (version 5.0) helped identify associated genes (referred to as candidate genes) and to further characterize the peak occupancy in the −5 kb region of TSS as well as all regions using differential binding analyses (Table 1). In absolute terms, however, for gene-activating H3Ac, the total number of peaks decreased by 52% in the diabetes-exposed group and 53% in the diet-exposed group respectively compared to controls. For H3K4me3, which is also an activating mark, the diabetes-exposed offspring acquired an additional 33% and diet-exposed offspring acquired an additional 73% more peaks. For repressive H3K27me3, diabetes-exposed and diet-exposed offspring acquired an additional 56% and 70% peaks, respectively (Figure 5). Compared to controls, the combination-exposed hearts had an additional 89% K4me3 peaks (Figure 5). 

**Figure 6:**
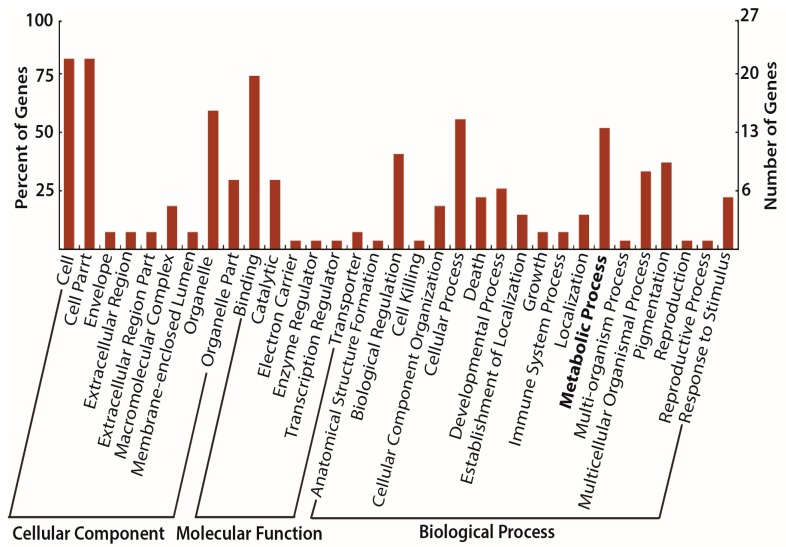
Gene ontology classification of genes with H3K4me3 enrichment histone modification due to high fat exposure. A representative gene ontology analysis of candidate genes corresponding to H3K4me3 peak enrichment in the diet exposed group (HC) is shown. Gene ontology of ‘metabolic process’ is chosen for the downstream analyses.

Differential binding analyses of 5 kb upstream of TSS revealed two and three H3Ac peak losses respectively in the diabetes-exposed and diet-exposed groups compared to controls (both, FDR < 0.1, Table 1, Supplementary Table S1). For H3K4me3 modification, 2 and 28 peaks were gained in diabetes-exposed and diet-exposed groups, respectively (all, FDR < 0.1, Table 1, Supplementary Table S1). Of note, the 28 differentially enriched H3K4me3 peaks in the diet-exposed group were annotated to 28 candidate genes, 15 of which were related to ‘metabolic process’ in the gene ontology analysis (Table 2 and Table 3, Figure 6). This indicates that in utero exposure to a maternal HF-diet induced epigenetic changes that contributes to fetal programming of cardiac metabolism. H3K27me3 showed a mixed response: six peaks were significantly gained for the diabetes-exposed and diet-exposed groups, while they also lost three and one peaks, respectively (Table 1). Differentially bound peaks in regions other than −5 kb of TSS are shown in Table 1, but were not further analyzed in gene ontology or disease ontology. 

### 3.4. Gene Promoter Occupancy of Diabetes and Diet Induced Differential Histone Modifications 

Only genes whose promoter region showed differential binding compared to the control group and returned hits against “metabolic process” in gene ontology are reported here. Enriched H3K4me3 peaks (enrichment fold difference with respective control indicated in parenthesis) occupied promoters of two functionally related genes, *heat shock protein 1a1* (*Hspa1a*) and *Hspa1b* in diabetes-exposed (6.54 fold, FDR = 0.02) and diet-exposed (5.62 fold, FDR < 0.001) groups, respectively (Table 2, Supplementary Table S2). Other noteworthy cardiometabolic genes associated with H3K4me3 peak gains in diet-exposed group include *ATP synthase mitochondrial Fo complex, subunit C3, subunit 9* (*Atp5g3*, 6.10 fold, FDR = 0.07); *Cytochrome P450, family 4, subfamily f, polypeptide 18* (*Cyp4f18*, 5.06 fold, FDR = 0.01); *autophagy associated transmembrane protein* (*EI24*, 2.79 fold, FDR = 0.02); *endoplasmic reticulum oxidoreductase beta* (*Ero1lb*, 6.66 fold, FDR = 0.002); *farnesyl diphosphate synthase* (*Fdps*, 1.9 fold, FDR = 0.06); *hexose-6-phosphate dehydrogenase* (*H6pd*, 6.75 fold, FDR < 0.001); *P450* (*cytochrome*) *oxidoreductase* (*Por*, 6.16 fold, FDR = 0.06); *solute carrier family 11, member 2* (*Slc11a2*, 6.7 fold, FDR < 0.001) and *tripartite motif containing 63, E3 ubiquitin protein ligase* (*Trim63*, 4.99 fold, FDR < 0.001) (Table 2).

For H3K27me3 repressive mark, loss of peak in the promoter region indicates a potential gain of gene function. Combination exposed offspring experienced H3K27me3 peak loss from the promoters of several candidate genes when compared to diabetes-only exposed offspring: *ATP binding cassette subfamily B member 9* (*Abcb9*, 5.6 fold, FDR = 0.02), *Atp5g2* (5.92 fold, FDR = 0.003), *lysine demethylase 6B* (*Kdm6b*, 5.37 fold, FDR = 0.08), *LIM domain binding 1* (*Ldb1*, 5.73 fold, FDR = 0.01), *nuclear receptor subfamily 4, group A, member 3* (*Nr4a3*, 3.41 fold, FDR = 0.004), *RuvB-like AAA ATPase 2* (*Ruvbl2*, 3.40 fold, FDR = 0.05), *solute carrier family 44, member 4* (*Slc44a4*, 6.17 fold, FDR < 0.001), *steroid sulfatase* (*microsomal*) *isozyme S* (*Sts*, 1.82 fold, FDR = 0.08), and *urocortin 2* (*Ucn2*, 2.61 fold, FDR = 0.06). On the contrary, combination-exposed offspring demonstrated a gain of H3K27me3 peaks (potential suppression of function) compared to the diabetes-only exposed group for *adenylate kinase 3* (*Ak3*, 1.95 fold, FDR = 0.09) and *signal transducer and activator of transcription 5B* (*Stat5b*, 3.76 fold, FDR = 0.09). Taken together, epigenetic changes induced by maternal HF-diet, diabetes, or a combination thereof were partially overlapping, but also distinct. 

### 3.5. Quantitative Association of Diet and Diabetes Induced Histone Modifications with Candidate Genes and QTLs Related to Cardiometabolic Functions

We carried out a quantitative genetic analysis using histone modification occupancy levels on the identified genomic regions focusing on cardiac or metabolic function related genes. Gene ontology revealed that *Hspa1a* and *Hspa1b* are associated with “primary metabolic process” against HF diet-induced H3K4me3 modification (log odd ratio 1.38, FDR = 0.06) (Table 3). Also, “positive regulation of cholesterol biosynthetic process” (FDR = 0.0295) was associated to *Fdps* and *Por* (Table 3 and Supplementary Table S2). Although, the candidate genes belonged to distant genomic regions, 15 of them overlapped with a total of 455 QTLs, primarily consisting of 88 QTLs for blood pressure (19.34%), 24 QTLs for body weight (5.27%), and 20 QTLs for serum cholesterol (4.40%) (Table 4). Particularly, *Fdps* overlapped with a total of 33 QTLs, of which 22 (66.67%) were related to blood pressure. *Atp5g3* overlapped with a total of 22 QTLs, of which 9 (40.91%) were linked to body weight. Similarly, *Por* and *Ero1lb* overlapped with a total of 12 and 19 QTLs, respectively, out of which 2 (16.67%) and 3 (15.79%) were related to serum cholesterol, respectively, (Table 4 and Supplementary Table S3), suggesting the putative role of HF diet in hypercholesterolemia. Further, enrichment of genes in disease ontology revealed that the *Hspa1a* gene was related to multiple CVD indications such as coronary disease, atherosclerosis, and acute coronary syndrome, as well as disease progression of systolic heart failure (Supplementary Table S4). *Hspa1b*, a close relative of *Hspa1a*, was associated to obesity, type 2 diabetes mellitus, and hyperlipidemia. Fold enrichment (using FunDO [42] on 20 April 2016) of these two genes against histone peak occupancy in diet and diabetes exposed pups comparing with the control pups were 17.099 against type 2 diabetes mellitus (FDR = 0.018). Further, *H6PD* was related to experimental diabetes mellitus and inborn errors on metabolism (Supplementary Table S4), supporting the potential role of prenatal HF diet either alone or in combination with maternal diabetes on the fetal metabolic programming of adult-onset diabetes and CVD. However, the presented data from this exploratory study is newborn cardiac-tissue specific. The proof of concept generated here will be reexamined in the future in other physiologically relevant tissues in a time dependent manner through adulthood of the offspring generation.

### 3.6. Gene Expression Validation of Selected Candidate Genes

**Figure 7:**
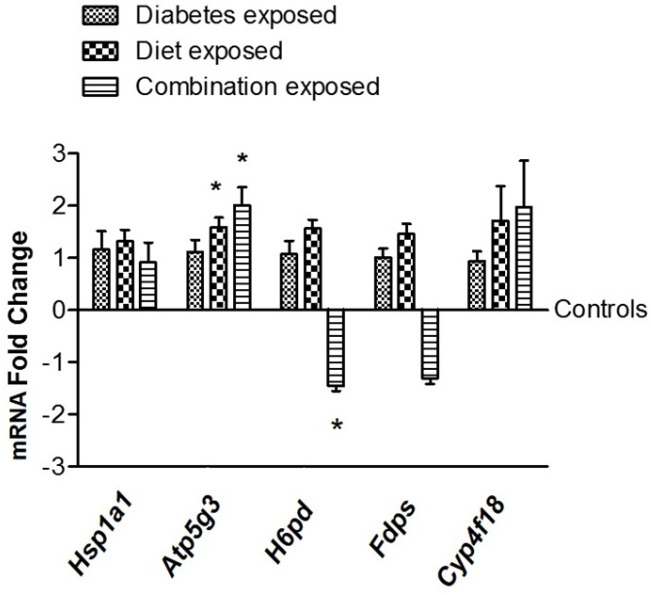
Gene expression validation of selected candidate genes with enriched H3K4me3 marks (compared to controls) on their promoters. Beta-2-microglobulin was used as a reference gene for normalizing gene expression levels. *n* = 4, * *p* < 0.05.

Gene expression validation was carried out for selected genes for which promoters gained epigenetic mark H3K4me3 (the highest differential peak enrichment was observed for this mark as shown in Table 1 and Table 2) compared to the respective control group. Gene selection was based on two criteria: those with higher levels of changes and also with functional relevance to our previously reported phenotype [11]. Due to the gains of the H3K4me3 mark in the diet exposed group for all genes and in the diabetes exposed group for one of the five selected genes (*Hsp1a1*), expression levels of these selected genes were anticipated to be upregulated in a corresponding manner. qRT-PCR results showed a trend for increased expression for all five genes in diet-exposed group as expected, with statistical significance achieved for *Atp5g3* only. Likewise, for *Hsp1a1* an increased expression trend was observed in the diabetes exposed group (Figure 7). A small sample size for newborn hearts (*n* = 4) may have, at least partially, contributed to the lack of statistical significance for some of the candidate genes. Although, the combination exposed group did not always show a synergistic impact on gene expression, this is in line with the observations for epigenetic marks (Figure 5). Additional investigation is needed in the future to understand the observations for the combination exposed group.

## 4. Discussion

Nutrient-mediated fetal metabolic programming due to a maternal HF diet and diabetes increases the risk of CVD at birth and throughout life. To our knowledge, this is the first report of genome-wide mapping of histone modifications in the cardiac tissue of offspring exposed to diabetic pregnancy and a prenatal HF diet, and the results suggest an epigenetic basis for fuel-mediated cardiac tissue reprogramming in utero. Furthermore, these results support our previous phenotypic observations that a maternal HF diet in conjunction with maternal diabetes causes mitochondrial dysfunction with impaired glycolysis, oxidative phosphorylation, and fatty acid oxidation in the exposed offspring heart at birth [11]. Cellular bioenergetics play a pivotal role in heart health, thus, understanding how these and additional candidate histone marks are causally linked to disease development could allow precise early-detection and prevention strategies for cardiometabolic dysfunction at birth and in later life. 

Histone modifications are typically reversible and precede more permanent gene-suppressive DNA methylation [22] and, for these reasons, are ideal candidates for preventive interventions. Among various known PTMs, methylation, and acetylation of histone H3 lysine residues have been the most thoroughly investigated in terms of biological implications. In this pilot work, we presented four major histone PTMs that involve H3 methylation and acetylation and are well-characterized in both human and rat [43,44,45]. Histone acetylation at all lysine residues opens and activates chromatin by responding to acetyl-CoA levels, which relates to the metabolic state of the host. Therefore, characterizing histone acetylation following metabolic perturbations, as in our in-utero model, may provide a mechanism for cells to initiate metabolic adaptations [46]. In contrast, site-specific methylations can have both an activating and a suppressive role in gene transcription and are among the most stable, frequently inherited PTMs. We chose to study the histone trimethylation marks at lysines 4 and 27, because we wanted to study both an activating (H3K4me3) and a repressive (H3K27me3) methylation mark. In particular, changes in H3K4me3 levels can have stable, long-lasting effects over several generations, even after the initial source of perturbation is no longer present [14,44,45,47]. 

Increased occupancy of H3K4me3 was observed in the promoter regions of various genes associated with metabolic stress and cardiac dysfunction in the HF-diet group, including those known to be involved in mitochondrial injury (*Atp5g3*, [48]), autophagy (*Ei24*, [49]), experimental diabetes mellitus (*H6pd*, [50], *Erol1b*, [51]), percentage body fat (*SLC11A2*, [52]), oxidation of inflammatory lipid substrates (*CYP4F*, [53]), and hypertrophic cardiomyopathy and chronic heart failure (*Trim63*, [54]). Of significant interest is increased H3K4me3 on promoter regions of the Atp5g3 gene. This gene encodes a portion of ATP synthase subunit c, part of the mitochondrial proton channel, and regulates both ATP synthesis and proton leak to influence the balance between cellular energy production from respiration and ROS production [55]. Using qPCR, we confirmed a significant cardiac-tissue specific increase in expression of *ATP5G3* in HF diet and combination-exposed newborns. Interestingly, hearts of offspring in these two groups also had significantly more lipid peroxidation [11]. Findings suggest a role for H3K4me3 of ATP5g3 in proton leak and oxidative stress in the developing heart. Our predicted gene expression results are further supported by proteomics literature, such as ATP synthase protein [56,57] and LIM-binding domain (LBD) protein [57,58] in a similar diabetes-induced rat model, and heat shock proteins (HSPs) in human patients with heart failure [59].

Two functionally related genes, *Hspa1a* and *Hspa1b*, also showed increased H3K4me3 occupancy in both the HF and diabetic groups. *Hspa1a* upregulation is linked with HF diet associated oxidative stress in patients with metabolic syndrome [60], correlates with HbA1c levels in maternal diabetes [61], and is proposed as an independent prognostic marker in patients with heart failure and cardiac arrest [59,62]. Our disease ontology analysis comparing HF-diet-induced, H3K4me3-occupied gene promoters with those of the control group linked *Hsp1a1* and *Hspa1b* activation with severe disease progression in systolic heart failure and CVD. Although qPCR did not confirm a tissue-specific increase in expression of *Hspa1a* in newborn offspring, H3K4me3 of *Hsp1a1* may still serve as a valuable prognostic indicator for heart health later in life. Following expression over time may be important for further understanding the role of heat shock proteins in developmentally programmed CVD following in utero exposure to maternal HF diet. 

In addition, enrichment of the *Por* and *Fdps* gene promoters with the H3K4me3 marks was correlated with “metabolic process” and “positive regulation of cholesterol biosynthesis process” in gene ontology analyses. *Por* is involved in cholesterol and bile acid synthesis [63], while *Fdps* activation is associated with hypercholesterolemia [64]. Enrichment was also found on the *Cyp4f18* promoter, a member of the cytochrome p450 complex involved in regulation of lipotoxicity. Interestingly, all of these annotated genes against differential H3K4me3 peak enrichment in the HF-diet group overlapped with ‘blood pressure’ QTLs, while most of them overlapped with ‘cholesterol synthesis’ QTLs. Newborn rat offspring exposed to a maternal HF diet had significantly increased cardiac lipid deposition [11]. While qPCR did not confirm an increase in tissue-specific Fdps expression possibly due to a smaller sample size, there was a trend towards increased cardiac expression of *Cyp4f18* in HF-diet exposed offspring. Future studies should investigate the role of epigenetics in cardiac lipotoxicity through these or other candidate genes.

Since the activating H3k4me3 and repressive H3K27me3 modifications coexist to form bivalent promoters in early life, promoters may also respond to developmental stimuli and change their histone modification state through H3K27me3 demethylation [47]. When we compared offspring exposed to late-gestation diabetes born to dams on control vs. a HF diet, a lysine demethylase enzyme-encoding gene, *Kdm6b*, was associated with significant H3K27me3 loss, suggesting potential upregulation of this enzyme. Other notable candidate genes with loss of H3K27me3 occupancy are *ABCB9* (associated with type 2 diabetes [65]); *Sts* (associated with HF diet and ob/ob models of obesity and type 2 diabetes [66]); and *Ucn2* (associated with heart failure patients [67]). Additionally, there was significant H3K27me3 loss in the promotor region of ATP5G2 which encodes another subunit in the ATP synthase proton pore. Loss of H3K27me3 could lead to tissue-specific upregulation and potentiate the effects of H3K4me3 induced ATP5G3 expression found in HF-diet exposed offspring. This may explain why combination-exposed offspring have the most severe mitochondrial dysfunction [11]. We also observed differential H3K27me3 marks in HF diet and STZ in combination when compared to STZ treatment alone (Table 2), suggesting HF diet has a major role in the combined treatment. 

Enrichment of H3K27me3 indicates potential gene silencing, and was observed in the HF-diet exposed offspring for *AK3*, a mitochondrial matrix protein that is highly expressed in the heart [68]) and negative regulation of adipogenesis (*STAT5B*, [69]). Expression of these genes may further explain the lipotoxic phenotype found in the hearts of HF diet-exposed newborn offspring [11]. In this context, we did not observe higher body weight in the HF-diet exposed newborns, although the hearts were larger and had significant lipid deposition [11]. Of note here is a previous study that used a similar rat model and found that HF-diet exposure in utero led to higher body weights and greater adiposity much later in the life of the offspring, in spite of having similar birth weights as control rats [70]. Histone modification predominantly H3K4 and H3K9 methylation closely links an oxidative stress pathway with underlying biochemical mechanisms of cardiovascular disease [71]. The fetal exposure to elevated glucose and free fatty acids likely generate cardiac mitochondrial reactive oxygen species, resulting in oxidative damage to the cardiomyocyte mitochondria. Although the mitochondrial numbers may increase as a part of compensation, overall mitochondrial efficiency goes down in diabetic hearts, leading to mitochondrial dysfunction playing a potentially pathogenic role in elevating risk of adult onset diabetes [72]. This is consistent with our previous findings [11] where we reported that HF diet exposure was associated with a higher copy number, but diabetes exposure alone (CD-STZ) was associated with a lower mitochondrial copy number than control. Overall, in the previous study we showed a significant interaction effect causing the poorest mitochondrial respiration in HF diet and STZ treatment combined group in the same rat model used in this study [11].

## 5. Conclusions

This study is the first to show that prenatal exposure to HF diet affects acetylated and methylated chromatin states in a site-specific manner along the rat genome in the offspring cardiac tissue and that these changes are present at birth. Together with others reporting liver-tissue-specific fetal metabolic programming [73,74,75], the results discussed here clearly demonstrate that epigenetic cues for metabolic disease risk are established in utero. These results further corroborate our previous reports on hyperinsulinemia and hyperlipidemia observed in rat offspring born to diabetic and/or HF-fed dams, particularly during pregnancy [11]. However, here we did not observe any synergy between prenatal exposure of a HF-diet and diabetes. The changes resulting from HF and diabetes exposure were distinct and the most hits against “metabolic process” in gene ontology analyses of differentially bound peaks came from H3K4me3 gains in the HF-exposed (HC) group when compared to the control (CC) group. Nevertheless, the results explain variable phenotypic findings in offspring from various groups. Moreover, data presented emphasize the critical role of the prenatal environment in epigenetically laying the foundation for the lifelong cardiometabolic health of offspring born following pregnancy affected by HF diet, diabetes, or both. Knowledge gained from this study will aid in a deeper mechanistic investigation of developmental programming of cardiometabolic disease and, if translatable, will set the foundation for the early identification and prevention of heart disease in at-risk infants. Understanding the role of diet-induced early epigenetic cues in the pathogenesis of disease is essential to halting the progression before symptoms ever develop and could be a key to decreasing the global burden of metabolic diseases.

## Figures and Tables

**Table 1:** Genome-wide, differential binding of enriched histone modification peaks.

Histone Modification	CC vs. CS (Gained/Lost Peaks)		CC vs. HC (Gained/Lost Peaks)		CS vs. HS (Gained/Lost Peaks)	
	All Region	Promoter Region	All Region	Promoter Region	All Region	Promoter Region
H3Ac	0/32	0/2	5/103	0/3	0/0	0/0
H3K4me3	8/6	2/0	449/11	28/0	1/6	1/0
H3K27me3	186/82	6/3	309/114	6/1	403/226	4/22

CC, controls; CS, diabetes exposed; HC, HF-diet exposed; HS, combination exposed.

**Table 2:** Genes corresponding to differentially bound histone peaks and with functional relevance to “metabolic process”.

Modification	Diets	Genes	Gene Names	Fold Change	FDR
H3Ac	CC vs. CS	Myrf	Myelin regulatory factor	−5.53	1.59 × 10^−2^
H3K4me3	CC vs. CS	Hspa1a	Heat shock protein 1A	6.54	2.49 × 10^−2^
		Hspa1b	Heat shock protein 1B	6.54	2.49 × 10^−2^
	CC vs. HC	Atp5g3	ATP synthase, subunit C3	6.10	7.63 × 10^−2^
		Cyp4f18	Cytochrome P450, polypeptide 18	5.06	1.27 × 10^−2^
		Ei24	EI24, autophagy associated transmembrane protein	2.79	2.42 × 10^−2^
		Ero1lb	Endoplasmic reticulum oxidoreductase beta	6.66	1.96 × 10^−2^
		Fdps	Farnesyl diphosphate synthase	1.92	5.66 × 10^−2^
		H6pd	Hexose-6-phosphate dehydrogenase	6.75	7.39 × 10^−2^
		Hspa1a	Heat shock protein 1A	5.62	3.76 × 10^−2^
		Hspa1b	Heat shock protein 1B	5.62	3.76 × 10^−2^
		Mapk8ip2	Mitogen-activated protein kinase 8 interacting protein 2	6.16	6.08 × 10^−2^
		Napsa	Napsin A aspartic peptidase	6.18	5.66 × 10^−2^
		Nfix	Nuclear factor I/X (CCAAT-binding transcription factor)	2.09	8.40 × 10^−2^
		Por	P450 (cytochrome) oxidoreductase	6.16	6.08 × 10^−2^
		Slc11a2	Solute carrier family 11, member 2	6.73	9.55 × 10^−2^
		Trim63	Tripartite motif containing 63	5.00	9.51 × 10^−2^
		Zim1	Zinc finger, imprinted 1	6.28	3.43 × 10^−2^
H3K27me3	CC vs. CS	Bmp8b	Bone morphogenetic protein 8b	2.25	3.03 × 10^−2^
		Gli1	GLI family zinc finger 1	2.77	6.51 × 10^−2^
		Rps29	Ribosomal protein S29	3.73	7.29 × 10^−2^
		Snapc3	Small nuclear RNA activating complex, polypeptide 3	4.51	7.29 × 10^−2^
		Ccdc126	Coiled-coil domain containing 126	−4.10	9.17 × 10^−2^
	CC vs. HC	Dstyk	Dual serine/threonine and tyrosine protein kinase	2.57	8.56 × 10^−2^
		Lhpp	Phospholysine phosphohistidine inorganic pyrophosphate phosphatase	5.63	1.69 × 10^−2^
		Zfp444	Zinc finger protein 668	−5.83	4.77 × 10^−2^
	CS vs. HS	Ak3	Adenylate kinase 3	1.95	9.12 × 10^−2^
		Stat5b	Signal transducer and activator of transcription 5B	3.76	8.66 × 10^−2^
		Abcb9	ATP binding cassette subfamily B member 9	−5.60	2.22 × 10^−2^
		Atp5g2	ATP synthase, mitochondrial Fo complex, subunit C2	−5.92	3.41 × 10^−2^
		B3gnt3	UDP-glcnac:betagal beta-1,3-*N*-acetylglucosaminyltransferase 3	−5.79	7.71 × 10^−2^
		Kdm6b	Lysine demethylase 6B	−5.37	7.80 × 10^−2^
		Ldb1	LIM domain binding 1	−5.73	1.10 × 10^−2^
		Metap1	Methionyl aminopeptidase 1	−5.58	2.46 × 10^−2^
		Nfic	Nuclear factor I/C	−4.58	3.65 × 10^−2^
		Nr4a3	Nuclear receptor subfamily 4, group A, member 3	−3.41	3.70 × 10^−2^
		Ruvbl2	Ruvb-like AAA atpase 2	−3.40	5.43 × 10^−2^
		Slc44a4	Solute carrier family 44, member 4	−6.18	2.79 × 10^−2^
		Snapc3	Small nuclear RNA activating complex, polypeptide 3	−4.17	4.90 × 10^−2^
		Sts	Steroid sulfatase (microsomal), isozyme S	−1.82	7.95 × 10^−2^
		Ucn2	Urocortin 2	−2.61	5.70 × 10^−2^
		Uqcrq	Ubiquinol-cytochrome c reductase, complex III subunit VII	−5.73	1.10 × 10^−2^

Bolded genes either gained activating H3K4me3 or lost repressive H3K27me3 mark. CC, controls; CS, diabetes exposed; HC, HF-diet exposed; HS, combination exposed.

**Table 3:** Gene ontology of identified genes in the HF-diet exposed group.

Gene	Enriched GO ID	Description of GO	Log 2 Odd Ratio	FDR
Atp5g3	GO:1901360	Organic cyclic compound metabolic process	1.96	0.0976
	GO:0044238	Primary metabolic process	1.38	0.0636
Cyp4f18	GO:1901360	Organic cyclic compound metabolic process	1.96	0.0976
	GO:0044248	Cellular catabolic process	2.87	0.0976
	GO:0044238	Primary metabolic process	1.38	0.0636
	GO:0016709	Oxidoreductase activity	6.13	0.0976
Ei24	GO:0030308	Negative regulation of cell growth	4.80	0.0726
Fdps	GO:0045542	Positive regulation of cholesterol biosynthetic process	8.68	0.0195
	GO:1901360	Organic cyclic compound metabolic process	1.96	0.0976
	GO:0044238	Primary metabolic process	1.38	0.0636
H6pd	GO:1901360	Organic cyclic compound metabolic process	1.96	0.0976
	GO:0044238	Primary metabolic process	1.38	0.0636
Hspa1a	GO:0008180	COP9 signalosome	6.31	0.0976
	GO:1901360	Organic cyclic compound metabolic process	1.96	0.0976
	GO:0044248	Cellular catabolic process	2.87	0.0976
	GO:0090083	Regulation of inclusion body assembly	8.09	0.0312
	GO:0044238	Primary metabolic process	1.38	0.0636
Hspa1b	GO:0008180	COP9 signalosome	6.31	0.0976
	GO:0043281	Regulation of cysteine-type endopeptidase activity involved in apoptotic process	4.43	0.0976
	GO:1901360	Organic cyclic compound metabolic process	1.96	0.0976
	GO:0044248	Cellular catabolic process	2.87	0.0976
	GO:0090083	Regulation of inclusion body assembly	8.09	0.0312
	GO:0044238	Primary metabolic process	1.38	0.0636
Mapk8ip2	GO:0044238	Primary metabolic process	1.38	0.0636
Napsa	GO:0044238	Primary metabolic process	1.38	0.0636
Nfix	GO:1901360	Organic cyclic compound metabolic process	1.96	0.0976
	GO:0044238	Primary metabolic process	1.38	0.0636
Por	GO:0045542	Positive regulation of cholesterol biosynthetic process	8.68	0.0195
	GO:0043281	Regulation of cysteine-type endopeptidase activity involved in apoptotic process	4.43	0.0976
	GO:0044248	Cellular catabolic process	2.87	0.0976
	GO:0044238	Primary metabolic process	1.38	0.0636
	GO:0016709	Oxidoreductase activity	6.13	0.0976
Slc11a2	GO:0043281	Regulation of cysteine-type endopeptidase activity	4.43	0.0976
	GO:1901360	Organic cyclic compound metabolic process	1.96	0.0976
	GO:0044238	Primary metabolic process	1.38	0.0636
Trim63	GO:0044248	Cellular catabolic process	2.87	0.0976
	GO:0044238	Primary metabolic process	1.38	0.0636

**Table 4:** Quantitative trait loci (QTL) related to metabolic processes that correspond to differentially bound histone peaks in the HF-diet exposed group.

Gene	QTL	QTL Count (Percent Total)						
		Blood Pressure	Body Weight	Glucose Level	Insulin Level	Insulin Dependent Diabetes Mellitus	Non-insulin Dependent Diabetes Mellitus	Serum Cholesterol
Atp5g3	22	7 (31.82%)	9 (40.91%)	1 (4.55%)				1 (4.55%)
Cyp4f18	14	3 (21.43%	3 (21.43%)		2 (14.29%)		1 (7.14%)	1 (7.14%)
Ei24	19	7 (36.84%)		2 (10.53%)	1 (5.26%)		2 (10.53%)	2 (10.53%)
Ero1lb	19	7 (36.84%)	1 (5.26%)	2 (10.53%)	1 (5.26%)		2 (10.53%)	3 (15.79%)
Fdps	33	22 (66.67%)	3 (9.09%)				2 (6.06%)	
H6pd	12	7 (58.33%)						2 (16.67%)
Hspa1a	3	1 (33.33%)	1 (33.33%)			1 (33.33%)		
Hspa1b	3	1 (33.33%)	1 (33.33%)			1 (33.33%)		
Mapk8ip2	12	6 (50.00%)				1 (8.33%)	2 (16.67%)	1 (8.33%)
Napsa	16	7 (43.75%)	1 (6.25%)	1 (6.25%)			2 (12.50%)	2 (12.50%)
Nfix	4	1 (25.00%)						1 (25.00%)
Por	12	6 (50.00%)		1 (8.33%)	1 (8.33%)	1 (8.33%)	1 (8.33%)	2 (16.67%)
Slc11a2	8	5 (62.50%)					1 (12.50%)	
Trim63	19	7 (36.84%)	3 (15.79%)				1 (5.26%)	4 (21.05%)
Zim1	6	1 (16.67%)	2 (33.33%)				1 (16.67%)	1 (16.67%)
Total	455	88 (19.34%)	24 (5.27%)	7 (1.54%)	5 (1.10%)	4 (0.88%)	15 (3.30%)	20 (4.40%)

## Supplementary Materials

The following are available online at www.mdpi.com/2072-6643/9/4/407/s1. These are large Excel Spreadsheets that do not convert to PDF format correctly. Table S1: Differential binding analyses for upstream and overlap-start peaks in different diet groups. Table S2: Gene ontologies of candidate genes with H3K4me3 modifications due to maternal high fat diet. Table S3: Quantitative trait loci (QTL) overlapped with candidate genes with H3K4me3 modifications due to maternal high fat diet. Table S4: Disease ontologies of candidate genes with H3K4me3 modifications due to maternal high fat diet.
